# Solid-State NMR and Impedance Spectroscopy Study of Spin Dynamics in Proton-Conducting Polymers: An Application of Anisotropic Relaxing Model

**DOI:** 10.1021/acs.jpcb.1c06533

**Published:** 2021-11-08

**Authors:** Vytautas Klimavicius, Laurynas Dagys, Vaidas Klimkevičius, Dovilė Lengvinaitė, Kęstutis Aidas, Sergejus Balčiu̅nas, Juras Banys, Vladimir Chizhik, Vytautas Balevicius

**Affiliations:** † Institute of Chemical Physics, Vilnius University, LT-10257 Vilnius, Lithuania; ‡ Department of Chemistry, University of Southampton, SO17 1BJ Southampton, U.K.; § Institute of Chemistry, Vilnius University, LT-03225 Vilnius, Lithuania; ∥ Institute of Applied Electrodynamics and Telecommunications, Vilnius University, LT-10257 Vilnius, Lithuania; ⊥ Faculty of Physics, St. Petersburg State University, 198504 St. Petersburg, Russia

## Abstract

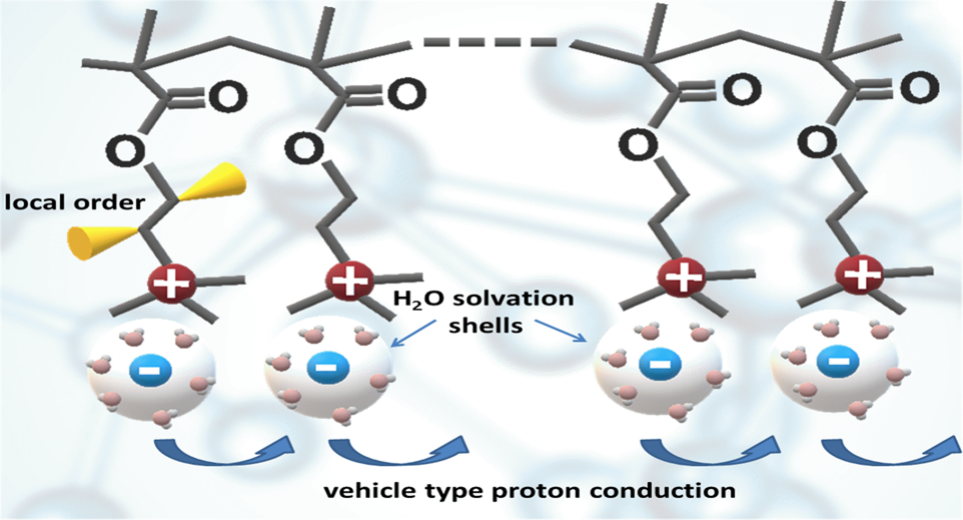

The ^1^H–^13^C cross-polarization (CP) kinetics in poly[2-(methacryloyloxy)ethyltrimethylammonium chloride] (PMETAC) was studied under moderate (10 kHz) magic-angle spinning (MAS). To elucidate the role of adsorbed water in spin diffusion and proton conductivity, PMETAC was degassed under vacuum. The CP MAS results were processed by applying the anisotropic Naito and McDowell spin dynamics model, which includes the complete scheme of the rotating frame spin–lattice relaxation pathways. Some earlier studied proton-conducting and nonconducting polymers were added to the analysis in order to prove the capability of the used approach and to get more general conclusions. The spin-diffusion rate constant, which describes the damping of the coherences, was found to be strongly depending on the dipolar I–S coupling constant (*D*_IS_). The spin diffusion, associated with the incoherent thermal equilibration with the bath, was found to be most probably independent of *D*_IS_. It was deduced that the drying scarcely influences the spin-diffusion rates; however, it significantly (1 order of magnitude) reduces the rotating frame spin–lattice relaxation times. The drying causes the polymer hardening that reflects the changes of the local order parameters. The impedance spectroscopy was applied to study proton conductivity. The activation energies for dielectric relaxation and proton conductivity were determined, and the vehicle-type conductivity mechanism was accepted. The spin-diffusion processes occur on the microsecond scale and are one order faster than the dielectric relaxation. The possibility to determine the proton location in the H-bonded structures in powders using CP MAS technique is discussed.

## 1 Introduction

The cross-polarization (CP) technique, often combined with magic-angle spinning (MAS), has been widely used in solid-state NMR studies for several decades.^1−4^ Typically, CP is used to enhance signals of less abundant spins (S) by using magnetization of abundant ones (I) with a larger gyromagnetic ratio. As CP is promoted by the dipolar I–S interactions that are intrinsically sensitive to internuclear distances, it plays a major role for probing short-range ordering and local dynamics.^5,6^ This technique can reveal fine aspects of structural organization in very complex solids, where other traditional methods work unsatisfactorily. Indeed, CP MAS kinetics, i.e., the dependence of the NMR signal intensity on the contact time for interacting spins, has revealed its capability to resolve fine structural effects as well as dynamics at the atomic level.^7,8^ The studies on spin diffusion and relaxation processes are very useful for understanding fine details of materials suitable for quantum information processing and of supramolecular aggregates for molecular electronics.^9,10^

It is well-known that the spin-diffusion processes are dominant in solid-state NMR dynamics. Traditionally, in the presence of strong heteronuclear dipolar interactions, e.g., between ^1^H and ^13^C, the kinetics of CP is described by the so-called I–I*–S model (I = ^1^H and S = ^13^C) combining a coherent term of the isolated I*–S spin pair and an incoherent term related to interactions with other protons in a thermal spin-bath in a phenomenological way.^7−9^ This model can qualitatively explain different build-up constants and general relaxation rates for different chemical groups. However, the major drawback arises in the chemical groups where interaction of the heteronuclear spin pair becomes comparable or weaker than the interaction with the surrounding spin reservoir. The model in such cases may not be sufficient to fit the spin diffusion and hence be unreliable. This can be fixed by readjusting the model with an appropriate approximation or a new model as well as by acquisition of very detailed CP MAS experiments.^8,11^

In this work, the CP MAS technique was applied to a series of synthetic polymers that can be categorized as smart polymers or “intelligent gels” that respond to external stimuli, which can be used in sensors, shape memory materials, and self-healing systems.^12^ The increasing interest to polymers and supramolecular electrolytes that are commonly regarded as clean energy fuel technology can be correlated with the necessity for the proton conductivity studies.^10^ Therefore, a set of solid-state methods sensitive to conductivity in combination with structural organization should be developed to meet the demand.

**Figure 1:**
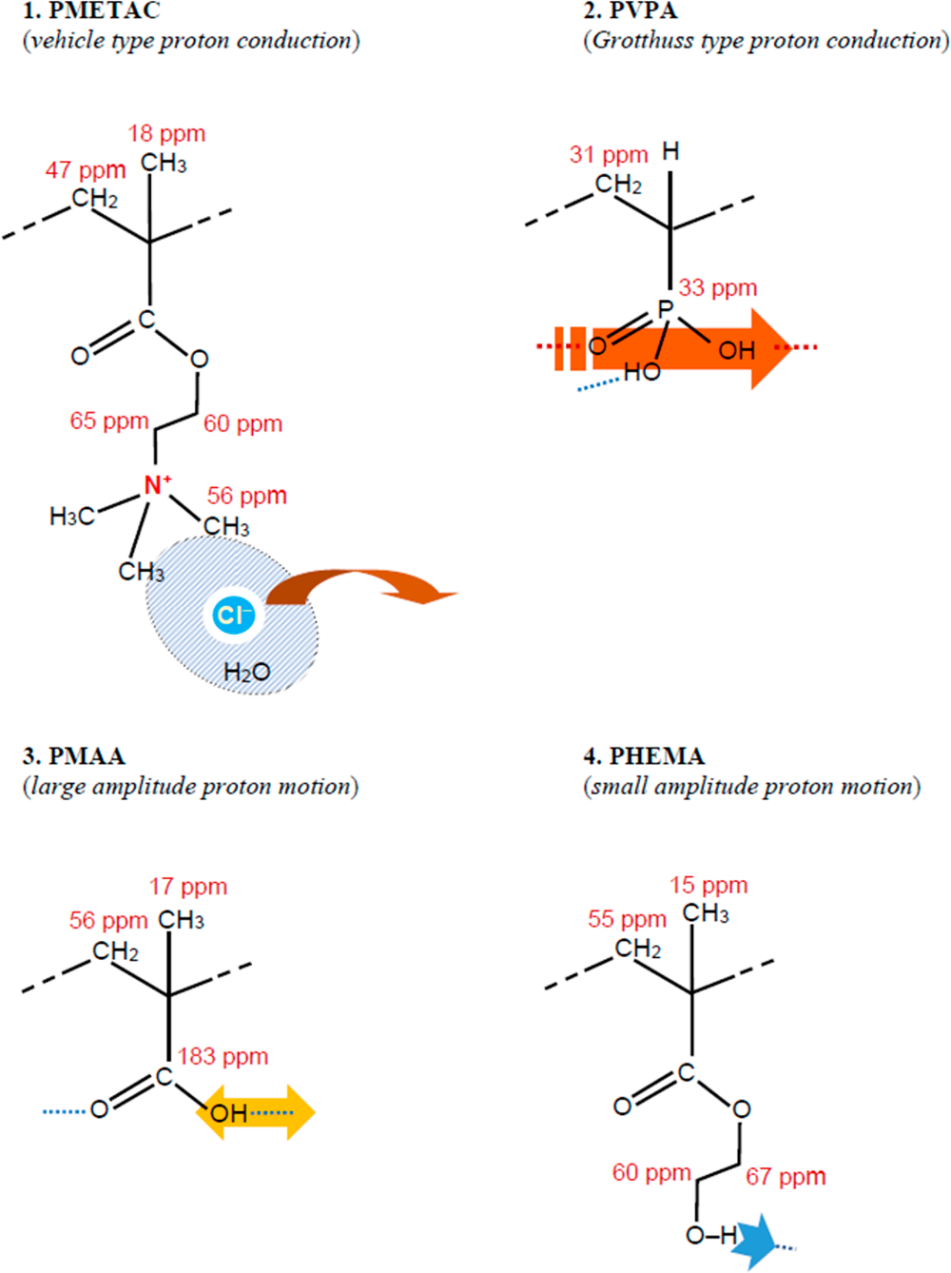
Simplified presentation of the studied polymers. The signal assignments and the chemical shifts for (2–4) are taken from refs (14−16). The experimental and calculated ^13^C CP MAS NMR spectra of the newly studied subject (1) are presented in Figure 2.

PMETAC (poly[2-(methacryloyloxy)ethyltrimethylammonium chloride]) is a polymer containing positively charged groups (quaternary ammonium groups) and Cl^–^ anions and thus could be utilized in the formation of anion conductive films.^13^ Here we invoke the CP MAS methodology to analyze the spin dynamics using the model developed by Naito and McDowell,^7^ which appropriately incorporates rotating frame spin–lattice relaxation mechanisms. In the present work this model was modified for spin clusters and adapted for powder samples under MAS conditions for the first time. In order to validate the capability of this approach and to obtain more consolidated view on the spin processes, some ^1^H → ^13^C and ^1^H → ^31^P CP MAS kinetics from our previous works, poly(2-hydroxyethyl methacrylate) (PHEMA),^14^ poly(metacrylic acid) (PMAA),^15^ and poly(vinyl phosphonic acid) (PVPA),^16,17^ were reprocessed by applying this method. The chosen systems are different with respect to hydrogen bonding (H-bonding) and proton conductivity features (Figure 1). PVPA is a confirmed proton-conducting polymer, where the Grotthus conductivity mechanism was deduced.^16−18^ Due to the presence of ions solvating water, the vehicle-type of proton conductivity is expected in PMETAC as well. We have successfully demonstrated that anisotropic relaxation during cross-polarization and adapted CP models well-match the predictions of impedance spectroscopy.^15,17−19^ Further, we have contrasted results with PMAA and PHEMA polymers, which are not proton conductors.^15^ This signifies that combination of these two methods are sufficiently developed to be used for new series of polymer conductors.

## 2 Experimental Section

### Monodisperse Polymers

Poly[2-(methacryloyloxy)ethyltrimethylammonium chloride] (PMETAC), poly(methacrylic acid) (PMAA), and poly(2-hydroxyethyl methacrylate) (PHEMA) (see Figure 1) with narrow molecular weight distribution were synthesized via radical addition–fragmentation chain transfer (RAFT) polymerization. More details on synthesis and characterization of PMAA, PHEMA, and PMETAC are given in refs (14), (15), and (20). Detailed synthesis procedure of PMETAC is provided in the Supporting Information.

Commercial poly(vinyl phosphonic acid) (PVPA), 99.9% purity, from Sigma-Aldrich, was used without further purification and treatment. The PMETAC samples were prepared under ambient conditions (further “wet” samples) and, in order to check the role of adsorbed water for proton conductivity process, were vacuum-dried for 1 day at room temperature (dried samples). The monitoring of the drying is presented in the Supporting Information (Figures S1–S3).

### NMR Measurements

The solid-state NMR experiments were carried out on a Bruker AVANCE III HD spectrometer using a 4 mm double resonance CP MAS probe. The experiments were performed at 298 K in a 9.4 T magnetic field using an Ascend wide bore superconducting magnet. The resonance frequencies of the ^1^H and ^13^C nuclei are 400.2 and 100.6 MHz, respectively. The samples were spun at the magic angle at the rate of 10 kHz using a 4 mm zirconia rotor. To fulfill one of the Hartmann–Hahn matching conditions in CP MAS experiments, rectangular (63 kHz and 66 kHz ^13^C RF field for “wet” and “dry” samples, respectively) variable contact time pulses were used. In the present work, all experiments were adjusted to fulfill the *n* = +1 condition. The CP MAS kinetics were recorded by varying the contact times from 50 μs to 10 ms in increments of 25 μs. All experimental details used for measuring 2–4 samples (Figure 1) and signal referencing are given in refs (14−16).

### The Impedance Spectroscopy

Powder was placed between two cylindrical brass electrodes and compressed with 0.24 MPa uniaxial pressure. Dielectric data were obtained by measuring the capacitance and loss tangent with an HP 4284 LCR-meter. Afterward, a flat capacitor model was implemented to calculate the complex dielectric permittivity. The measurements were carried out in 120–450 K temperature and 10^2^–10^6^ Hz frequency ranges. The temperature was measured with a Keithley 2700 multimeter and a 100 Ohm platinum resistor. Cooling and heating were done at a 1 K/min cooling/heating rate.

### The FTIR Absorption Spectroscopy

The IR absorption spectra were measured using an FTIR spectrometer Alpha (Bruker Optik GmbH) equipped with single reflection diamond ATR module. Spectra were measured in the spectral region from 4000 to 400 cm^–1^ with 4 cm^–1^ spectral resolution. Sixty-four interferograms were averaged and Fourier transformed into a spectrum by applying a three-term Blackman–Harris apodization function and a zero filling factor of 2.

## 3 DFT Calculations

**Figure 2:**
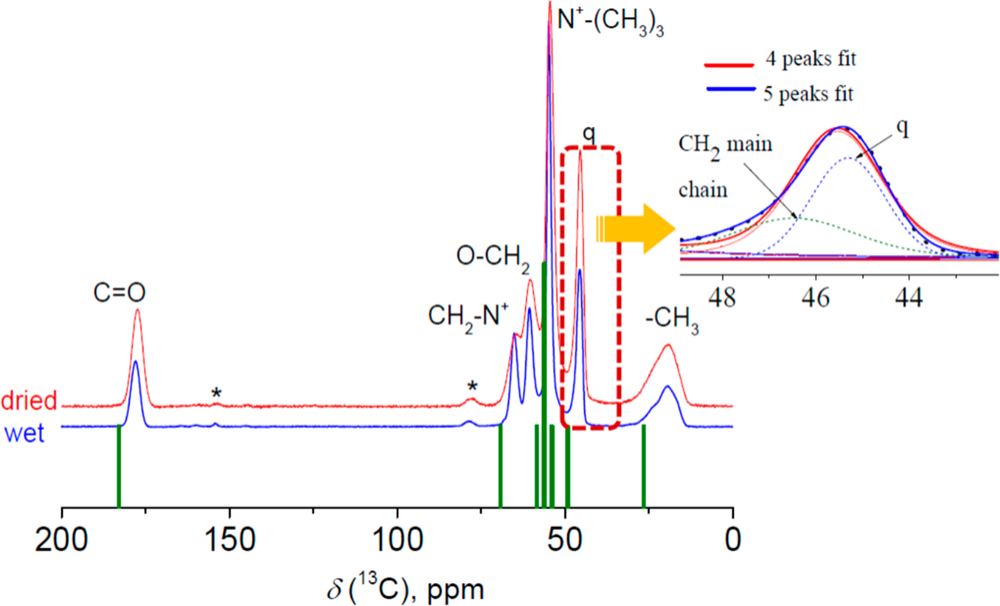
Experimental and calculated ^13^C CP MAS spectra of PMETAC. The presence of CH_2_ main chain signal that is overlapped with quaternary carbon peak is confirmed by the contour shape analysis using 4 and 5 peaks fit of signal group in the range of 30–70 ppm range.

Calculations of NMR isotropic magnetic shielding constants for the ^1^H and ^13^C nuclei in PMETAC have been performed using the density functional theory (DFT). The B3LYP exchange–correlation functional in combination with the 6-311G** basis set was used for geometry optimization of isolated fragments of PMETAC. The magnetic shielding tensors have been calculated *in vacuo* by using the modified hybrid functional of Perdew, Burke, and Ernzerhof (PBE0) along with the 6-311++G(2d,2p) basis set. The gauge-including atomic orbital (GIAO) approach^21^ was used to ensure gauge invariance of the results. The Gaussian 16 program^22^ was used for all our calculations. The ^1^H and ^13^C chemical shifts were obtained by subtracting the computed isotropic shielding constants of PMETAC from the corresponding shielding constants of TMS which are taken from ref (23). Our approach was proven to be adequate in various cases earlier. For example, satisfactory agreement between calculated and experimentally measured ^1^H NMR chemical shifts was obtained not only for molecular systems involved in strong H-bonding but also for rather “inert” species, e.g., CH_3_ protons (see refs (23) and (24), and references therein). We have analyzed isolated fragments of PMETAC composed of 2 monomers in order to support the assignment of experimentally observed signals (Figure 2). The calculated ^13^C NMR chemical shifts are shown together with the experimental ^13^C CP MAS spectrum in Figure 2. As seen in Figure 2, computational results agree with the experimental data fairly well.

## 4 Theoretical Spin Dynamics Model

Many of widely used theoretical CP kinetic models that exhibit the coherent oscillatory behavior of intensity originate from the pioneer work of Müller et al.^25^ This is the so-called I–I*–S model.^4,11,26^ The system is treated as a strongly coupled I*–S spin pair immersed in a spin-bath consisting of the remaining I spins (I = ^1^H and S is either ^13^C or ^31^P spins in the present work). There is assumed that only one spin I* interacts with the I-spin bath (or infinite energy reservoir of I spins), which is described in a phenomenological way. The kinetics of the CP signal intensity *I*(*t*), i.e., its dependency on the contact time (*t*), in the case of the isotropic spin diffusion is expressed as
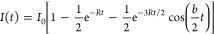
1where the parameter *R* is the spin-diffusion rate constant. The cosine-oscillation frequency is *b*/2, i.e., 1/2 of the dipolar splitting, which depends on the gyromagnetic ratios (*γ*_I_, *γ*_S_) of the two interacting nuclei (I and S), the distance *r* between them and the angle θ between the **r** vector and the magnetic field:
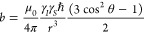
2This model was modified by Naito and McDowell^7^ taking into account the anisotropy of spin-diffusion and spin–lattice relaxation processes in the rotating frame:
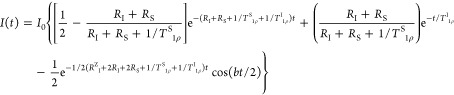
3Here *R*^*Z*^_I_ and *R*_I_ are the spin-diffusion rates of a particular I spin, and *R*_S_ is that of a particular S spin; the rotating frame spin–lattice relaxation is treated in a phenomenological way introducing the time constants *T*^I^_1ρ_ and *T*^S^_1ρ_ for the I and S spins, respectively.

Recently, the I–I*–S model and spin diffusion were studied very thoroughly by Hirschinger and Raya by solving quantum mechanical Liouville–von Neumann equation using various approaches and the formalism of spin-diffusion superoperator.^4,8,27^ For a fast fluctuating I-spin bath, the spin-diffusion superoperator for the reduced density operator σ̂ can be written as
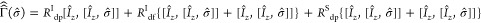
4where *R*^I^_dp_ and *R*^I^_df_ are the homonuclear spin-diffusion rate constants of the I* spin and *R*^S^_df_ is that (heteronuclear) of the S spin. The rate constants *R*^I^_df_ and *R*^S^_df_ are associated with the flip-flop terms of the homonuclear (I–I*) and heteronuclear (I–S) dipolar Hamiltonians, respectively, whereas *R*^I^_dp_ acts on the damping of the coherence.^27^

To our knowledge, eq 3 in its rigorous form, i.e., the one with the complete set of relaxation pathways for I and S spins, was used to describe the CP kinetics in single crystals in the static regime (no MAS) only, e.g., in the case of l-alanine.^7^ In many other studies the simplified eq 3 with asymptotes *T*^I^_1ρ_, *T*^S^_1ρ_ → ∞, have been used.^11,28^ Furthermore, as the dipolar splitting *b* is an “angular” function, the proper angular averaging has to be carried out in order to apply this equation to powder samples. Following ref (29), the angular averaging (AA) is carried out as
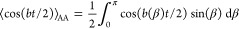
5

Then the eq 3 can be rewritten in the notations of Hirschinger and Raya, replacing *R*_I_ + *R*_S_ → *R*^*Σ*^_df_ = *R*^I^_df_ + *R*^S^_df_, *R*^*Z*^_I_ → *R*^I^_dp_ and carrying out the angular averaging, as
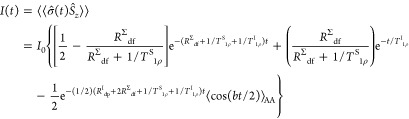
6

For CP MAS experiments, when the HH matching conditions ω_1I_ – ω_1S_ = *nω*_MAS_ are fulfilled for *n* = ±1 (used in the present work), the AA procedure has to be carried out on the cos(*b*_±1_*t*/2) oscillation that contains the spherical components of the **b**-tensor
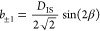
7where *D*_IS_ is the heteronuclear I–S dipolar coupling constant (*D*_IS_ = (1/2π)(μ_0_/4π)*γ*_I_*γ*_S_(*h*/2π)/*r*^3^, in Hz) and β is the polar angle between **r** vector and the MAS rotor axis.^27,29^ This can be done analytically^29^ using the series of Bessel functions *J*_*n*_:
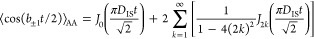
8

The effects of truncation of infinite Bessel series (Σ^∞^) were checked. It was deduced that the truncation already at *k* = 3 had no significant influence on the precision of calculations due to very steep suppression (∼1/(2*k*)^2^) of the contributions from *J*_2*k*_(*x*) of higher order.

The Naito and McDowell model (eq 6) considers a C–H spin pair. In order to handle CH_2_ and CH_3_ groups it should be modified for the spin clusters I*_n_–S. This can be done introducing the parameter λ that is related to the cluster size *n*.^11^ However, λ depends on the group mobility and therefore must be adjusted by the fitting of experimental and calculated curves.^11,26^ The theoretical model contains many variable parameters. Even using the parametrization without λ (eq 6) it is difficult to ensure the correct convergence of the nonlinear fitting flow to the “true” minima for some data sets with higher experimental “noise”. Trying to reduce the number of variable parameters it can be supposed that for dilute spins S (^13^C in the present case) should be *T*^I^_1ρ_ ≪ *T*^S^_1ρ_. This was deduced in the Naito and McDowell work for l-alanine.^7^ Also note that the approach *T*^S^_1ρ_ → ∞ is often set for ^13^C–^1^H pairs in other organic compounds.^11^ Therefore, the 1/*T*^S^_1ρ_ was neglected for the materials studied in the present work.

Then, if the I*–S coupling constant is much larger than the spin diffusion rate constants (|*b*| ≫ *R*^I^_df_, *R*^I^_dp_) and *R*^I^_df_ + *R*^S^_df_ > 1/*T*^I^_1ρ_ is valid, the modified eq 6 can be written as
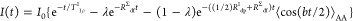
9

The nonlinear curve fitting was carried out using eqs 7–9 and applying the Levenberg–Marquardt algorithm implemented in the Microcal Origin and MathCad packages.

## 5 Results and Discussion

**Figure 3:**
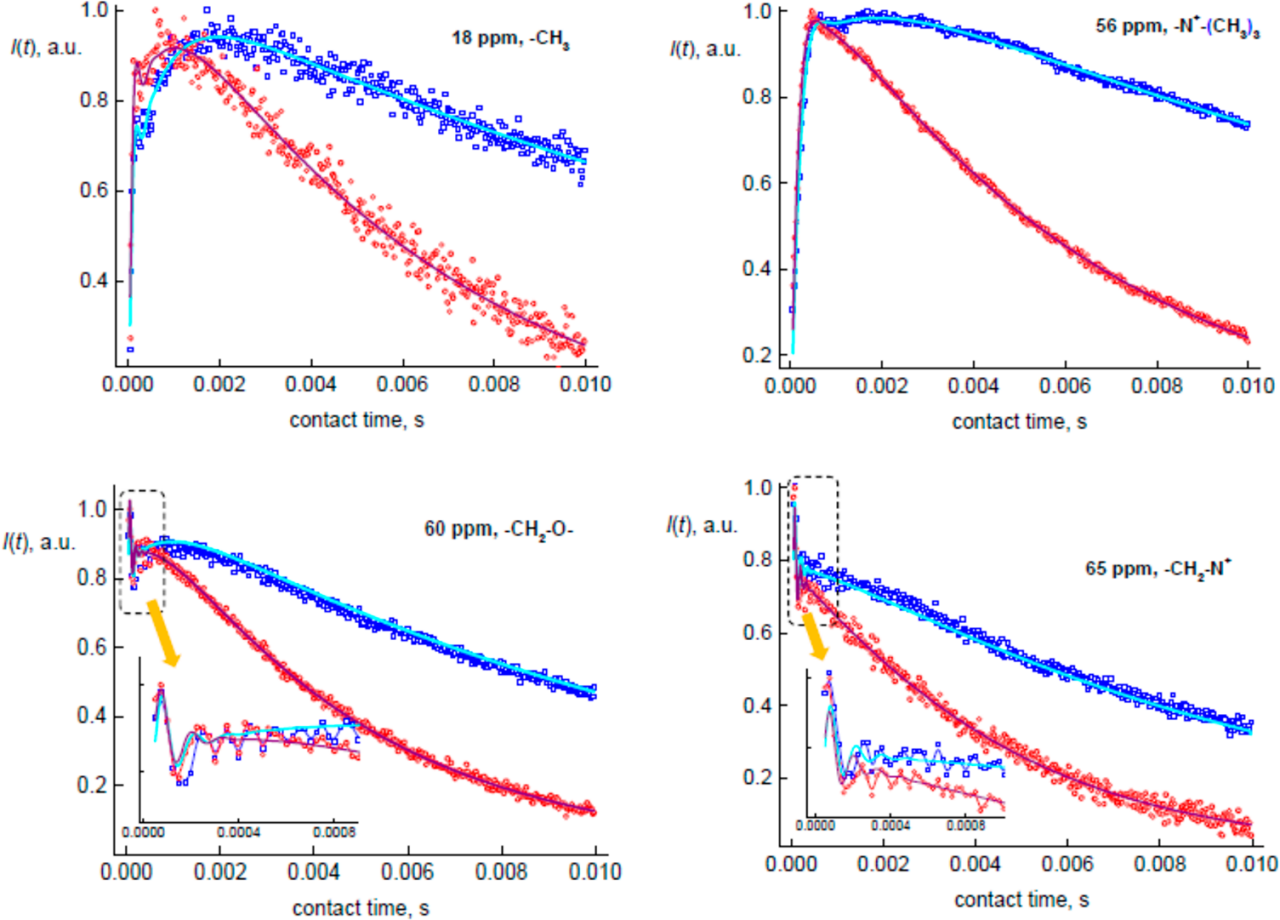
Experimental ^1^H → ^13^C CP MAS kinetic curves in wet (blue) and dried (red) PMETAC for various spin sites (see Figure 1) at 10 kHz MAS rate, processed using the anisotropic relaxing spin dynamics model with the implemented AA (eqs 7–9, solid lines). The nonlinear fitting results are presented in Table 1.

The ^1^H → ^13^C CP MAS kinetic build-up curves for various spin sites in PMETAC are presented in Figure 3. Two separate approaches were used to analyze the CP processes between adjacent (directly bonded) and remote (distanced more than one chemical bond) spins in various materials.^14−17^ In the case of adjacent spins the digital averaging is performed more accurately; namely, the coherent cos(*bt*/2) oscillations (eq 1) are summed by weighting cosine values by the fraction of spin pairs with a set of spatial parameters that corresponds to the oscillation frequency *b*/2. Such a routine implicates the angular and distance averaging. However, the shape of the dipolar splitting distribution is usually unknown and can be complex for soft disordered solids. Various shapes have to be tested for the practical use in the processing of the experimental CP kinetic curves, and the most proper one has to be chosen.^30,31^ However, the physical meanings of some parameters are not well-defined. The CP between remote spins was analyzed in the frame of the anisotropic spin-diffusion model improved by the thermal equilibration in the proton bath and allowing the asymptotic regimes *b* → 0 (weak interactions) and *n* → ∞ (a large bath).^5,16^ However, such a purely phenomenological model is based on some assumptions which lack rigorous quantum mechanical description of spin dynamics. For comparison, the processing results using these two approaches (cos-averaging and thermal equilibration model) were taken from refs (16) and (17). These results together with the results obtained for PVPA are presented in the Supporting Information (Figure S4).

The relaxing Naito and McDowell spin dynamics model modified for spin clusters and adapted for powders upon MAS (eqs 7–9) produces the same or even a better fit of experimental curves. It is a surprising finding because, in comparison with cos-averaging and thermal equilibration models, fewer variable parameters are used. The values of fitted parameters and some statistical criterions are given in Table 1. Hence, the anisotropic relaxing model with the implemented AA appeared to be the uniform approach that allowed precise description of CP kinetics for adjacent as well as for remote spins in a rigorous way.

**Table 1:** Fitted Model Parameters (Eqs 7–9) and the Local Order Parameters (*S*, Eq 10) for Various Spin Sites (Figure 1) in PMETAC^a^

NMR peak	λ	*R*^Σ^_df_, s^–1^	*R*^I^_dp_, s^–1^	*T*^I^_1ρ_, s	*b*, Hz (no AA)	*D*_IS_,^b^ Hz (AA)	*S*	*R*^2^/χ^2^ (%)
1. PMETAC								
–CH_3_	0.50	1480	9160	0.021	4510	9080	0.39	0.988/
18 ppm	0.42	1521	10020	0.0065	4170	8450	0.37	3.4
–N^+^(CH_3_)_3_	0.23	690	7160	0.022	1150	2320	0.10	0.998/
56 ppm	0.21	1380	8340	0.0063	1200	2410	0.10	1.6
–CH_2_–O	0.22	1350	16800	0.012	12200	21030	0.91	0.998/
60 ppm	0.26	1280	18370	0.0045	13050	22230	0.97	1.7
–CH_2_–N^+^	0.1	620	15080	0.010	12070	20080	0.87	0.993/
65 ppm	0.25	540	15100	0.0038	13000	22240	0.97	2.4
2. PHEMA								
–CH_3_	0.47	860	13700	0.015	4640	9250	0.42	0.981/
15 ppm								2.3
–CH_2_– main chain	0.38	1250	37300	0.011	13170	25100	1.00(9)	0.996/
55 ppm								1.4
–CH_2_–OH	0.41	1080	20300	0.0098	8110	11340	0.49	0.997/
60 ppm								1.4
–CH_2_–O–	0.39	840	22600	0.0088	11250	20030	0.87	0.994/
67 ppm								2.0
3. PMAA								
–CH_3_	0.39	920	11700	0.0067	4200	8400	0.37	0.981/
17 ppm								4.2
–CH_2_–	0.35	1730	43900	0.0050	10330	20950	0.91	0.993/
56 ppm								3.1
–COOH	0.50	2010	1930	0.0069	1860	3570		0.961/
183 ppm								5.8
4. PVPA								
–CH_2_–	0.50	1400	33600	0.0026	9140	17400	0.76	0.996/
31 ppm								2.8
–POOH	0.50	2160	6090	0.0026	1900	4370		0.999/
33 ppm								1.5

a The numbers in the upper rows correspond to the “wet” sample, the lower rows–to the dried one), PHEMA, PMAA and PVPA; *R*^2^ is the correlation coefficient and χ^2^ is the sum of weighted squares of deviations.

b The *D*_IS_ values are comparable with the dipolar splitting obtained directly from the Fourier transform over the experimental *I*(*t*) and rescaled by the factor of  because HH matching for *n* = ±1 was fulfilled (for PMETAC see Figure S5 in the Supporting Information and refs (14), (15), and (17) for other polymers).

The calculations implementing the angular averaging (eq 8) provide more accurate evaluation of the spin-diffusion rates (*R*^I^_dp_ and *R*^*Σ*^_df_) and the heteronuclear I–S dipolar coupling constants (*D*_IS_). For a powder, the destructive interference of the orientation-dependent coherences is expected to contribute significantly to the decay of the transient oscillations. Indeed, it was deduced that *R*^I^_dp_(with AA) = (0.78 ± 0.02) and *R*^I^_dp_(without AA), whereas the angular averaging had no effect on the *R*^*Σ*^_df_ values: *R*^*Σ*^_df_(with AA) = (1.1 ± 0.1) and *R*^*Σ*^_df_(without AA) (see the Supporting Information, Figure S6).

**Figure 4:**
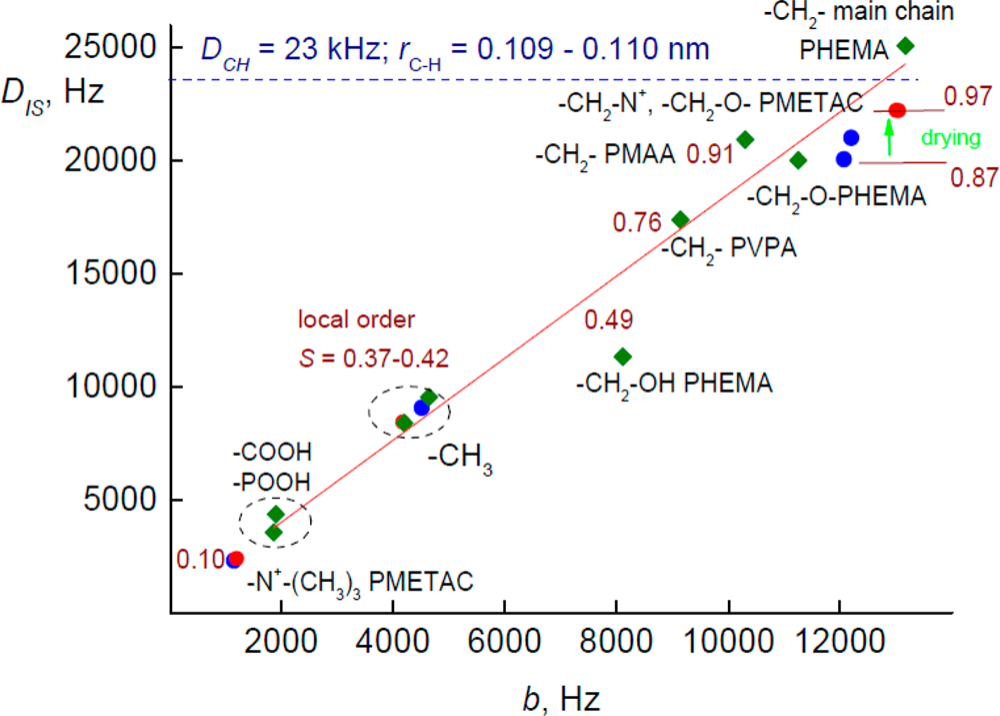
*D*_IS_ vs *b* plot. The *b* values are deduced from the model calculations without AA (Table 1). The calculated values of the local order parameter *S* (eq 10) are given for each spin site. The points that correspond to wet and dried PMETAC are indicated in blue and red, respectively.

The angular averaging effect is clearly seen in the *D*_IS_ vs *b* plot using the *b* values that follow from the calculations with no AA (Table 1, Figure 4). Roughly speaking, the AA procedure leads to the rescaling *D*_IS_ ≈ (1.8 ± 0.1)*b*. The accurate *D*_IS_ values are important for elucidating the site-resolved dynamic disorder associated with the local mobility of different functional groups or molecular segments. The local order parameter *S* was calculated in the well-known way^32,33^ as
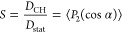
10where α is the angle of the instantaneous orientation of the dipole–dipole coupling tensor with respect to the symmetry axis of fast motion^32^ or the polar angle between the internuclear vector **r**_IS_ and the end-to-end vector of the polymer chain.^33^ The static constant *D*_stat_ for the ^13^C–^1^H dipolar coupling was taken 23.0 ± 0.3 kHz for the “frozen” C–H bond that corresponds to *r*_C–H_ ∼ 1.09–1.10 Å (eq 2).

The calculated *S* values for each site in the studied polymers are presented in Figure 4. The local order in the main chains and the proton motion in the H-bonds (Figure 1) are mutually coupled: the order parameter values for −CH_2_– sites in the main chains decrease as *S* ≈ 1.00(9) (PHEMA, i.e., weak H-bonds via O–H groups distanced from the main chain), 0.91 (PMAA, a large amplitude H-bond proton motion, however, no proton conductivity^15^), and 0.76 (PVPA, proton conductor^17,18^).

Two factors that can cause a marked error determining *D*_IS_ and *S* values have to be mentioned: (i) the *D*_IS_ values can be slightly overestimated due to the effect of radiofrequency field (RF) inhomogeneity on CP MAS. Indeed, the RF mismatch of 5–6% can cause the deviations in the dipolar splitting of 1–4 kHz;^3,34,35^ (ii) the fast MAS limit was not satisfied for some ^13^C–^1^H spin sites with the strongest spin couplings (>10 kHz). This could explain *D*_IS_ = 25.1 kHz deduced for the most rigid main chain in PHEMA (Table 1) and thus *S* > 1 to be caused by these factors.

The anisotropic relaxing spin dynamics model (eq 9) was applied for a series of powdered compounds under MAS condition. In the case of adjacent ^1^H–^13^C spins, the spin–lattice relaxation in the rotating frame for protons is relatively fast (*T*^I^_1ρ_ ∼ 10^–2^ to 10^–3^ s, Table 1). The drying of PMETAC reduces the rotating frame spin–lattice relaxation time constants *T*^I^_1ρ_ significantly (ca. one order).

**Figure 5:**
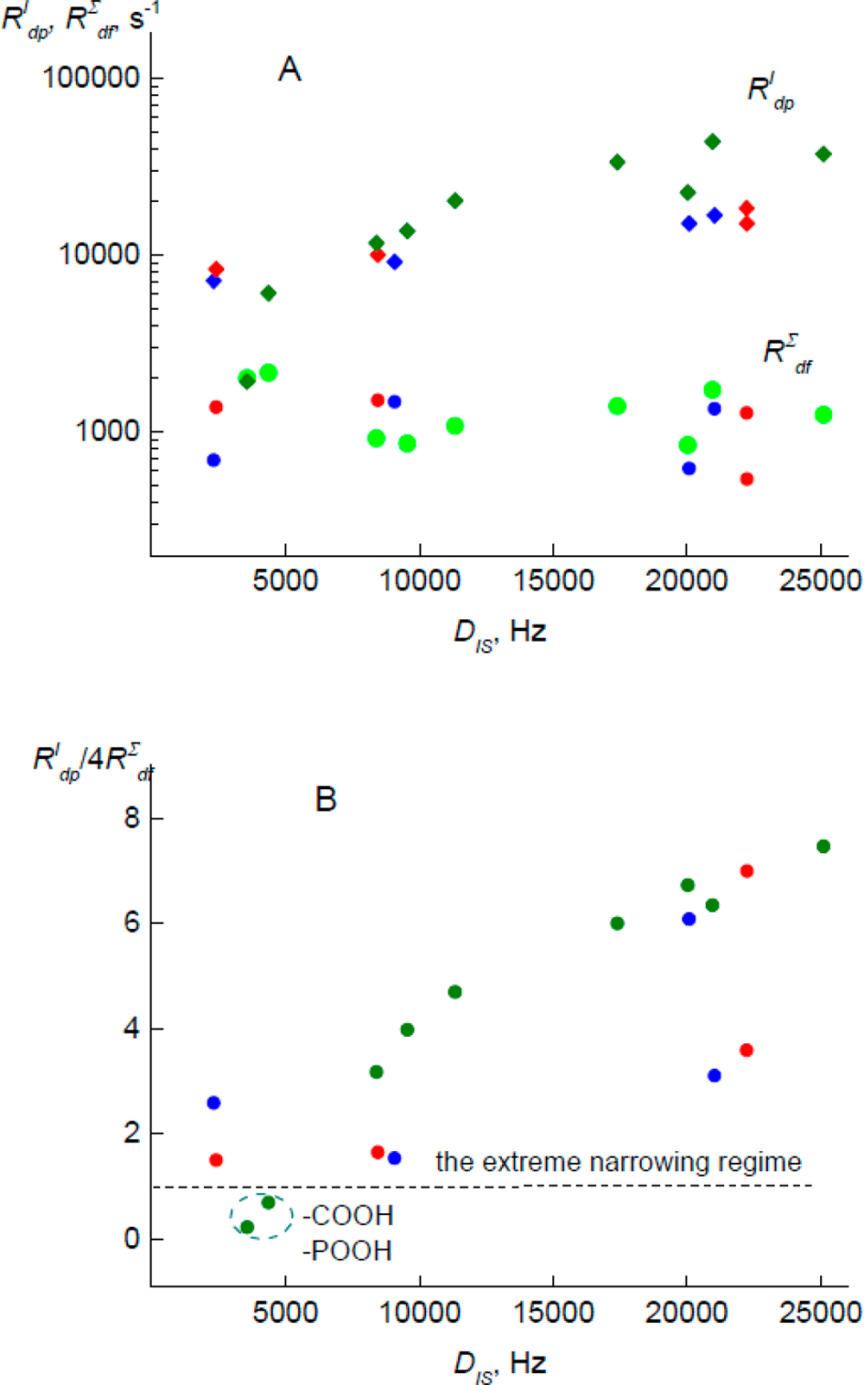
(A) Spin-diffusion rate constants *R*^I^_dp_ (rhombs) and *R*^Σ^_df_ (circles) vs dipolar coupling constants. (B) Anisotropy of spin diffusion. More comments are given in the text. The points that correspond to wet and dried PMETAC are indicated in blue and red, respectively.

The spin-diffusion rates *R*^I^_dp_ and *R*^*Σ*^_df_ obtained by the fitting of the experimental data and the model are presented in Figure 5. It is obvious that the drying of PMETAC scarcely influences the spin-diffusion rates in this polymer. The physical meaning of *R*^I^_df_ and *R*^S^_df_ are associated with the flip-flop term of the homonuclear (I–I*) and heteronuclear (I–S) dipolar Hamiltonian and allow the complete thermal equilibration with the bath.^8,27^ The total rate of *R*^*Σ*^_df_ = *R*^I^_df_ + *R*^S^_df_ processes was found to be weakly depending on the heteronuclear I–S dipolar coupling constant *D*_IS_ (Figure 5A). The *R*^I^_dp_ damps the coherences and drives the system toward internal quasi-equilibrium.^2^*R*^I^_dp_ was found to strongly depend on the dipolar coupling (probably ∼*D*_IS_^2^). It is often observed that *R*^I^_dp_ is much higher than *R*^I^_df_; i.e., the I–I* interactions reveal a high anisotropy.^4^ Such behavior (*R*^I^_dp_/*R*^I^_df_ ≫ 1) was observed also in the studied materials (Figure 5B).

**Figure 6:**
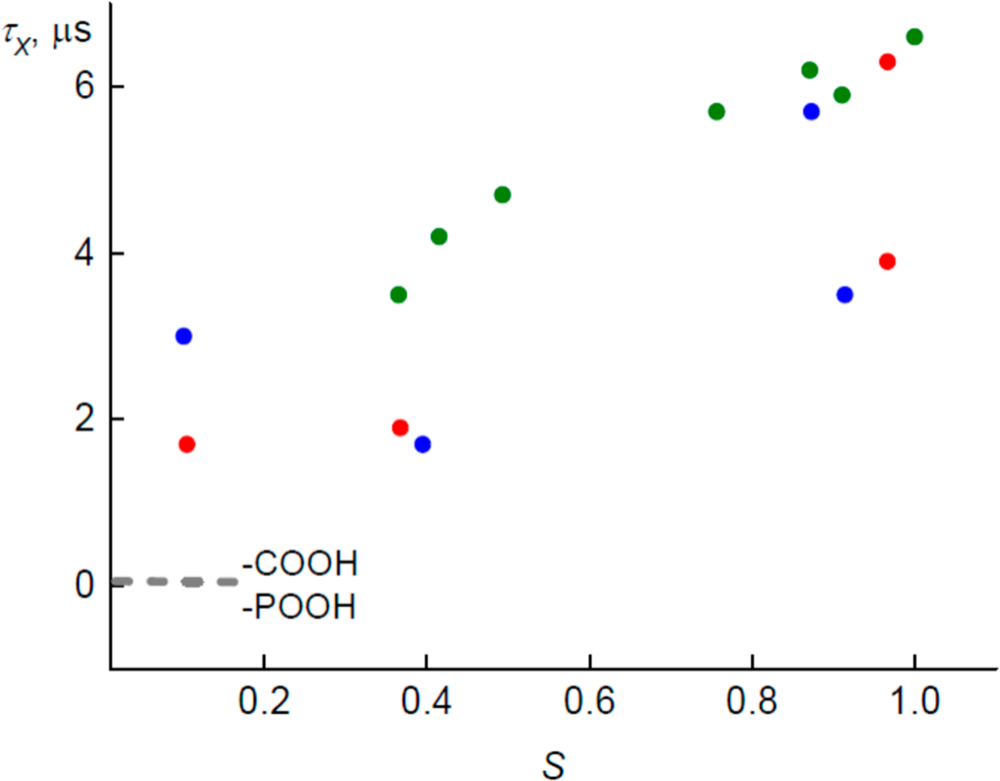
Dependency of the correlation time of the I-spin bath in the rotating frame *τ*_X_ on the local order parameters *S*. The points that correspond to wet and dried PMETAC are indicated in blue and red, respectively.

Random molecular motions cause fluctuations of the spin interactions and, hence, of the local fields. The rate of these fluctuations can be described by the correlation time of the I-spin bath in the rotating frame *τ*_X_. In the extreme narrowing regime (ω_1I_*τ*_*X*_ ≪ 1) the limiting ratio of spin-diffusion rates reaches the value *R*^I^_dp_/*R*^*I*^_df_ = 4.^4,36^ This condition was fulfilled only for −COOH in PMAA and −POOH in PVPA groups (Figure 5B). The *τ*_X_ was calculated using the formula derived in ref (4) (see the Supporting Information). The calculated *τ*_X_ values fairly well correlate with the local order parameters *S* (Figure 6).

**Figure 7:**
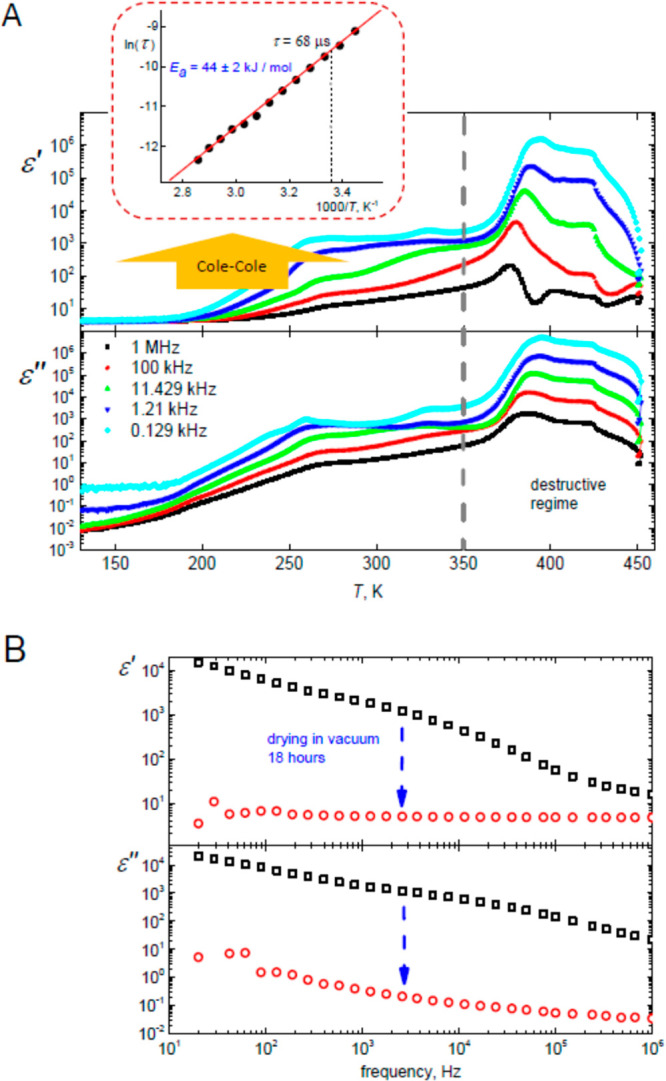
(A) Temperature dependences of real (ε′) and imaginary (ε″) parts of the complex dielectric permittivity of PMETAC at various frequencies. The dependence of the dielectric relaxation time (τ) obtained by Cole–Cole processing is presented as Arrhenius plot in the inset. (B) Disappearance of proton conductivity and relaxation upon drying in a vacuum. More comments are given in the text.

The extreme cases are −COOH and −POOH groups in PMAA and PVPA. The correlation time for them can be in the nano- or even picoseconds range. Such high mobility is credibly related with a large amplitude of proton motion along the H-bonds and proton conductivity in those polymers (Figure 1). In summary, the random motions and the fluctuations in the proton spin baths in all studied polymers run in the time scale of microseconds and are one order faster than the dielectric relaxation (τ ∼ tens of microseconds, Figure 7). It has to be noted, the fast MAS limit was not satisfied for some ^13^C–^1^H spin sites with the strongest spin couplings (>10 kHz). The *R*^I^_dp_ and *D*_IS_ values can be strongly influenced by RF inhomogeneities, especially, if the fast MAS limit approximation is not satisfied. This factor can artificially cause the strong increase of *R*^I^_dp_ with *D*_IS_ (Figure 5) and would distort the dependency of the correlation time of the I-spin bath (Figure 6). It is suspected that the refinement of the experiment should lead to the lower values of *R*^I^_dp_ and *D*_IS_. A simple modeling using eqs S1 and S2 (Supporting Information) has shown that then the *τ*_X_ values should be even lower than those presented in Figure 6. Hence, the statement that *τ*_X_ is one order faster than τ is correct. Nevertheless, it has to be noted that the correlation time of the I-spin bath (Figure 6) was calculated by assuming that the secular approximation is valid for all spin sites.

The problem of secularity has to be discussed in more details. Equations 1, 3, 6 and 9 are valid at the Hartmann–Hahn condition only if two secular approximations are satisfied: (i) the applied RF fields are much stronger than I*–S coupling (ω_1I_, ω_1S_ ≫ |*b*|); (ii) the I*–S coupling constant is much larger than the spin diffusion rate constants (|*b*| ≫ *R*^I^_df_, *R*^I^_dp_).^4,7,25^ Therefore, the physical legitimacy to use eq 9 for weakly interacting spins (*b* ≈ *R*^I^_df_, *R*^I^_dp_ or even less) is in a certain doubt. This situation is observed for some spin sites in the present work. Alvarez et al.^37^ have obtained the analytical nonsecular solution of the generalized Liouville–von Neumann equation for arbitrary values of the homonuclear spin-diffusion rate constants, however, for a static sample (no MAS) and neglecting the I–S interaction with environment (*R*^S^_df_ = 0). In ref (27) it was shown that this equation has a semi-nonsecular analytical solution when |*b*| ≫ |*R*^I^_df_ – *R*^S^_df_|. However, it was derived for nonrelaxing spin systems (*T*^I^_1ρ_, *T*^S^_1ρ_ → ∞). To avoid the effects of spin–lattice relaxation in the rotating frame, the ^1^H → ^31^P CP MAS kinetics has been measured for the nanostructured calcium hydroxyapatite and the seminonsecular model^27^ has been applied.^38^ The results have been compared with those obtained by the secular approach (eq 1). It was concluded that the secular model can be used with certain reservations also in the case |*b*| ≈ *R*^I^_dp_.

However, note, in the pioneering work of Ernst et al.^25^ it was mentioned that for a spin-diffusion rate *R* of the same order of magnitude as the dipolar coupling *b*, the rate constant of the nonoscillatory part is strongly dependent on the ratio *R*/*b*, whereas the decay constant of the oscillatory part is to a large extent independent of *b*. The strong dependence of *R*^I^_dp_ on the dipolar coupling *D*_IS_ observed in the series of polymers (Figure 5) contradicts this outlook. Most probably, this can be due to the properties of spin diffusion in the studied systems fall too far out from the frames of the secular approximation.

The proton mobility was studied by impedance spectroscopy. The temperature dependences of real (*ε*′) and imaginary (*ε*″) parts of the complex dielectric permittivity ε* = *ε*′ – i*ε*″ are shown in Figure 7A. The conductivity tail in the wet PMETAC is observed below 1 kHz, and the dielectric relaxation is spread over the frequency range from 10^3^ to 10^5^ Hz. Note, at the heating above ∼350 K, the thermal destruction of this polymer was observed. The experimental dielectric spectra become not reproducible in this regime. The drastic changes in the ^13^C NMR spectra (new peaks appear) allow us to state that the irreversible chemical reactions run at high (350–450 K) temperatures. Therefore, further experiments were restricted below 350 K. In order to check the role of adsorbed water in proton conductivity processes, the samples were vacuum-dried at room temperature. The effect of drying on the dielectric spectra is presented in Figure 7B.

**Figure 8:**
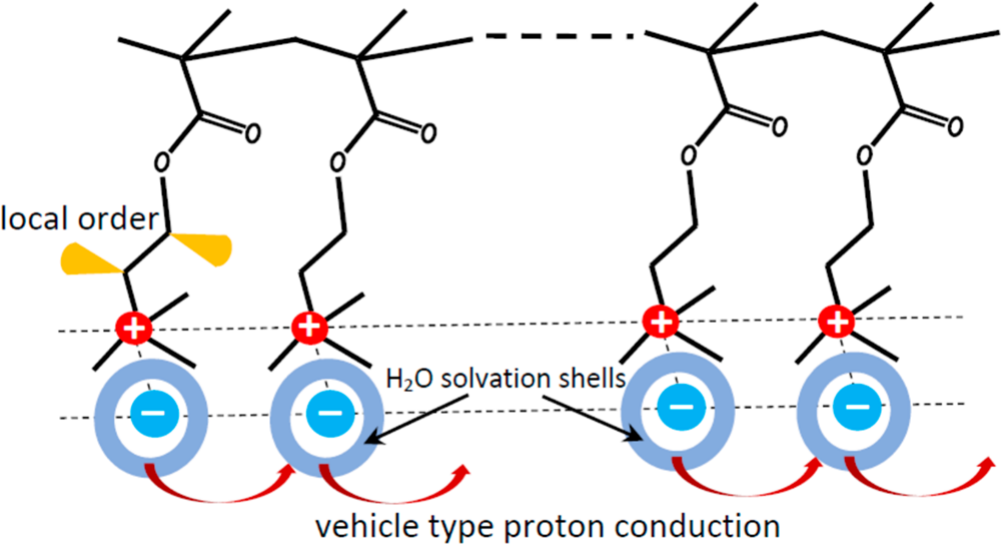
Schematic representation of proton conductivity in PMETAC. The cones symbolize the local disorder around −CH_2_– segments due to the restricted internal motion (bending, rocking, etc.) of C–H bonds.^14,15^ More comments are given in the text.

The disappearance of proton conductivity is seen upon drying (Figure 7B). This definitely indicates that in PMETAC these processes are related to water. The most probable location sites of water molecules are in the solvation shells surround Cl^–^ anions, as the tight access of water to the positively charged quaternary ammonium is sterically restricted (Figure 8).

Two main types of proton conduction via water can be distinguished: the vehicle-type mechanism and the Grotthuss mechanism.^39^ In the vehicle-type mechanism, proton bonds to water molecule forming H_3_O^+^ ion that moves through the medium in a diffusion-style process. The Grotthuss mechanism can be subdivided into two types. One is called as structural diffusion, where the water fluctuation is indispensable because it can overcome the rates determining reorientation steps.^39^ According the second type of Grotthuss mechanism, protons move from oxygen to oxygen via the *hop-and-turn* steps by simultaneously breaking and forming H-bonds.^15,18,39^ The water mobility or sometimes even the presence of water is not required.

The dielectric relaxation (Figure 7A) was approximated using the Cole–Cole function, and the low frequency data were recalculated to conductivity and approximated with Jonscher’s power law (see the Supporting Information). The obtained temperature dependencies of relaxations times and DC conductivity were further fitted using Arrhenius law.

The activation energy calculated for dielectric relaxation *E*_a_ = 44 ± 2 kJ/mol (the inset in Figure 7A) is much higher than for the bulk water (19–20 kJ/mol). It is also higher than those deduced for water diffusion in other media, e.g., 8–14 kJ/mol in brain white matter,^40^ ∼31 kJ/mol in hardened cement paste^41^ and ∼38 kJ/mol in some polyimide films.^42^ However, the presence of ions drastically influence diffusion process. The ^1^H NMR study of water diffusion in sodium chloride solutions have shown that the activation energy can increase up to 40 kJ/mol or even higher, depending on temperature and concentration.^43^ The higher activation energy in PMETAC can be understood accepting the vehicle mechanism, i.e., the main proton carriers are H_3_O^+^ ions that move via the heterogeneous medium that consists of the organic framework and ionic N^+^ and Cl^–^ substructure (Figure 8). Moreover, the presence of relatively narrow (∼180 Hz) peak in the ^1^H MAS NMR spectrum at 4.5 ppm confirms a high mobility of water and other −OH containing species in wet PMETAC (Supporting Information, Figure S1).

The activation energy determined for the conductivity *E*_a_ = 59 ± 6 kJ/mol (Supporting Information, Figure S7) gets between the values 45 and 65 kJ/mol obtained for as-prepared and annealed PVPA.^17,18^ As in PVPA there are no other mobile species than −POOH protons, the proton conductivity is realized via two-steps *hop-and-turn* Grotthuss mechanism for proton migration: (1) displacement of a proton along a hydrogen bond; (2) transfer of the proton to another oxygen with formation of a new H-bond.^15,17,18^ The coincident activation energies sustain an opinion that discussions based on only *E*_a_ values may not be precise distinguishing both (Grotthuss and vehicle-type) mechanisms, and further supporting data are required.^39^ Indeed, the border between the vehicle-type and Grotthuss structural diffusion type mechanisms looks rather thin.

The conductivity and relaxation processes disappear upon drying (Figure 7B). It is interesting to note that the drying causes the polymer hardening. It is reflected in the changes of the local order deduced in ^1^H–^13^C CP MAS kinetics experiments. This effect is most clearly pronounced on the CH_2_ groups located in the PMETAC branches near the ionic pairs, i.e., CH_2_–N^+^ and CH_2_–O (Figure 1). The simplified visualization of local disorder can be done using the cone with semiangle θ_0_ that covers the restricted diffusion of vector **r**_IS_(*t*) joining the interacting spins I and S. The amplitude of internal motion (bending, rocking, twisting, etc.) is qualitatively visualized by the cone semiangle θ_0_. Such cones are schematically represented in Figure 8 for CH_2_–N^+^ and CH_2_–O spin sites. The local order parameter *S* (eq 10) is related to θ_0_ as *S* = cos θ_0_ (1 + cos θ_0_)/2.^44,45^ Then the increase of *S* upon drying from 0.87 to 0.97 (Figure 4) corresponds to the cone shrinkage—the semiangle θ_0_ decreases from 25° to 12°. However, the drying does not influence the internal dynamics and disorder of–CH_3_ groups in PMETAC (Figure 4).

Finally, it is interesting to note the results obtained for the H-bonded moieties in PVAA and PVPA (Figure 1). In the current treatment the asymptotic *b* → 0, which was used to describe the CP kinetics in remote and thus weakly interacting spin systems,^5,16^ was not involved. The spherical tensor components *b*_±1_ and the coupling constants *D*_IS_ (eq 7) were included among the variable parameters and determined by the curve fitting. The *D*_IS_ values of 3570 and 4370 Hz for −COOH and −POOH deduced applying AA (Table 1) correspond to the internuclear ^1^H···^13^C and ^1^H···^31^P distances of ∼2.04 and 2.23 Å, respectively. It is well-known that the direct and the most precise information on the proton position in the H-bond structures in the solid state is obtained by single-crystal neutron diffraction.^46,47^ Though relatively large single crystals are required, this is not feasible in many cases. Therefore, it would be a breakthrough if CP kinetics indeed provide complementary information on the hydrogen localization and proton transfer reactions in advanced materials of powder or gel form, e.g., amorphous or nanostructures materials, supramolecular aggregates, etc.

## 6 Concluding Remarks

1. The anisotropic relaxing spin dynamics model^7^ was modified and applied for a series of powdered polymers under MAS conditions. This approach allowed us to describe CP kinetics for adjacent as well as for remote spins in a unified way.
2. In the cases of adjacent ^1^H–^13^C spin pairs the spin–lattice relaxation for protons in the rotating frame is relatively fast (*T*^I^_1ρ_ ∼ 10^–2^ to 10^–3^ s). The fluctuations in the proton spin baths in the studied polymers run in the time scale of microseconds and are one order faster than the dielectric relaxation (tens of microseconds).
3. The model applied for a series of spin systems provides more details on the spin diffusion: the *R*^I^_dp_, which acts on damping the coherences and driving the system to the internal quasi-equilibrium, was found strongly depending on the dipolar coupling constant *D*_IS_; the total rate *R*^Σ^_df__,_ = *R*_Idf_ + *R*_Sdf_ associated with the flip-flop terms and the incoherent thermal equilibration with the bath, was found to be independent or very weakly depending on the heteronuclear coupling. The polymer drying, i.e., the switching-off the proton conductivity, scarcely influences the spin-diffusion rates.
4. The drying causes the polymer hardening that reflects the changes of the local order parameters deduced from the ^1^H–^13^C CP MAS kinetic experiments. This can be explained by drying that removes the electrostatic screening action of water solvation shells and thus the strengthening of ionic substructure.
5. A cautious optimism concerning the possibility to determine the proton location in the H-bonded structures in powders using CP MAS technique can be imparted. To confirm this rigorously, the CP MAS kinetics studies have to be carried out over a series of H-bonded systems that have precisely determined H-bond geometry, e.g., by neutron diffraction.

## Supporting Information Available

Details of synthesis; monitoring of drying of PMETAC in NMR, FTIR, and impedance spectra; monitoring of the PMETAC drying kinetics; comparison of various CP MAS kinetic models; Fourier transform (FT) over the time dependence of the oscillating part of CP intensity; calculation of the correlation time of the I-spin bath in the rotating frame; rate correlation; processing of impedance spectroscopy data; temperature dependence of direct current conductivity (PDF)
