# Supplementary material for: Solid-State NMR and Impedance Spectroscopy Study of Spin Dynamics in Proton-Conducting Polymers: An Application of Anisotropic Relaxing Model
Source: J Phys Chem B. 2021 Nov 8;125(45):12592–602. doi: 10.1021/acs.jpcb.1c06533 (PMC8607415; doi:10.1021/acs.jpcb.1c06533)
Supplement: Supplementary file 1 — jp1c06533_si_001.pdf [file jp1c06533_si_001.pdf]

Supporting information for publication

Solid-State NMR and Impedance Spectroscopy Study of Spin Dynamics in Proton-Conducting Polymers: An Application of Anisotropic Relaxing Model

Vytautas Klimavicius[1], Laurynas Dagys[2], Vaidas Klimkevičius[3], Dovilė Lengvinaitė[1], Kęstutis Aidas[1], Sergejus Balčiūnas[4], Juras Banys[4], Vladimir Chizhik[5], Vytautas Balevicius[1]*

[1]Institute of Chemical Physics, Vilnius University, LT-10257 Vilnius, Lithuania

[2]Department of Chemistry, University of Southampton, SO17 1BJ, Southampton, UK

[3]Institute of Chemistry, Vilnius University, LT-03225 Vilnius, Lithuania

[4]Institute of Applied Electrodynamics and Telecommunications, Vilnius University, LT-10257 Vilnius, Lithuania

[5]Faculty of Physics, St Petersburg State University, 198504 St Petersburg, Russia

Detailed synthesis procedure of PMETAC

METAC aqueous solution (5.19 g, 4.15 g of the monomer METAC, 20 mmol), 84 mg of the initiator 4,4′-Azobis(4-cyanovaleric acid) (0.3 mmol), and 262 mg of freshly synthesized RAFT CTA[20] (0.9 mmol) were placed into a round-bottom flask containing 35 g of a mixed solvent MeOH/H_2O (70/30 v/v). Concentration of the monomer in the solution was 15%, molar ratio of the monomer to the CTA and the initiator was $[M]_0/[CTA]_0/[I]_0$ = 100/4.5/1.5. The reaction mixture was bubbled for 20 min with argon and then stirred for 8 h at 70 °C. The product was precipitated by pouring the reaction mixture to 10-fold excess of acetone and dried in a vacuum oven at 40 °C until constant weight. Yield of slightly yellow pMETAC was 5.11 g (98.5%).

Monitoring of drying of PMETAC in NMR, FTIR and impedance spectra

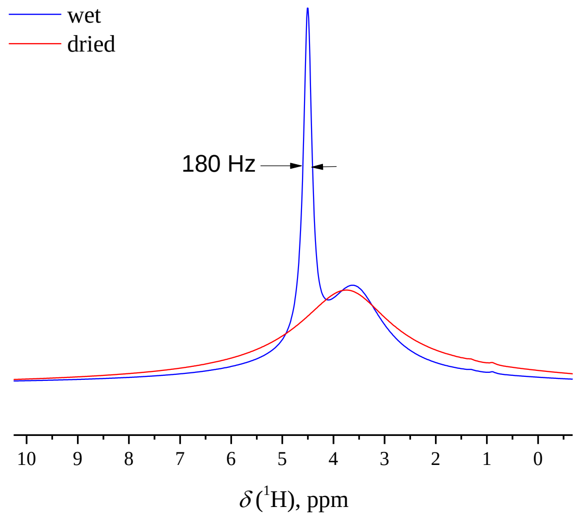

Figure S1. The ^{1}H MAS NMR spectra of PMETAC samples prepared at ambient conditions and vacuum dried for one day at room temperature.

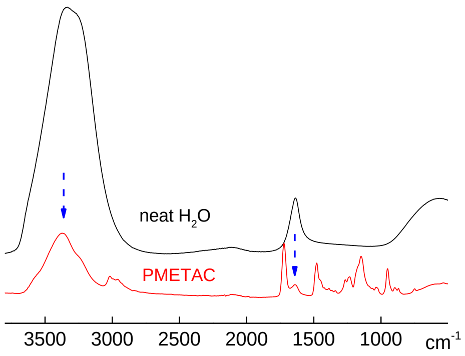

Figure S2. FTIR ATR spectrum of PMETAC under ambient conditions. The spectrum of the neat water is added to attribute the bands. The spectra were recorded on BRUKER-ALPHA spectrometer.

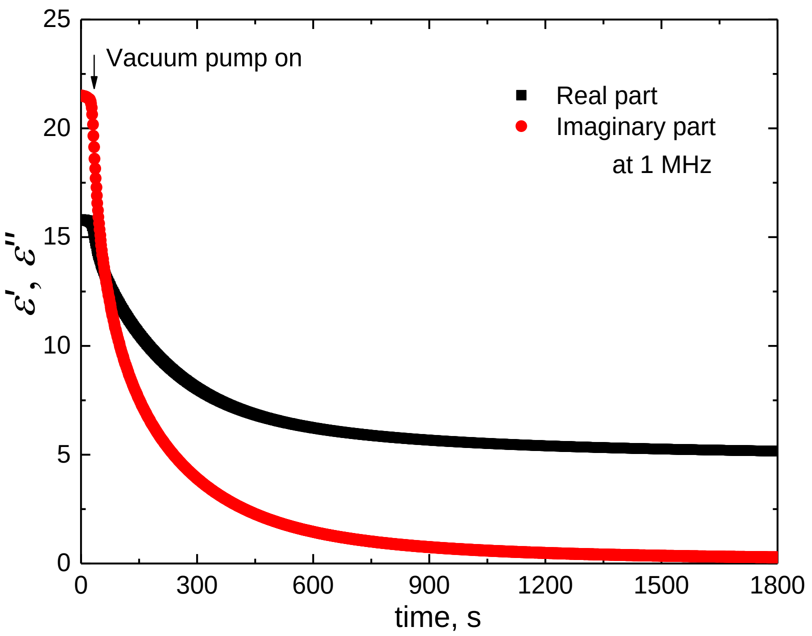

Figure S3. The monitoring of the PMETAC drying kinetics on the dielectric permittivity at 1 MHz.

The comparison of various CP MAS kinetic models

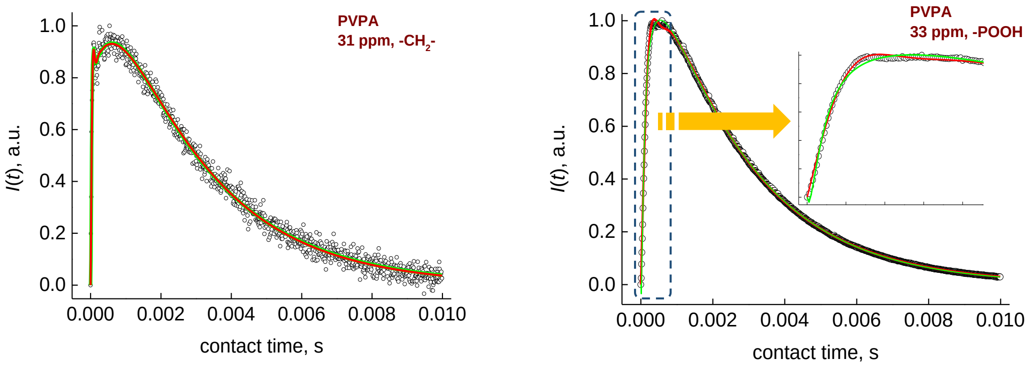

Figure S4. Experimental $^{1}H \rightarrow ^{13}C$ and $^{1}H \rightarrow ^{31}P$ CP MAS kinetic curves (circles) for CH_2 and –POOH spin sites in PVPA (Figure 1) at 10 kHz MAS rate, processed using the anisotropic relaxing spin dynamics model with the implemented AA (eqs 6 - 8, red line); the fitting results using other CP MAS approaches (cos-averaging for CH_2 and thermal equilibration for –POOH, respectively) have been taken from Refs 16,17 and shown by the green lines.

The Fourier transform over the time dependence of the oscillating part of CP intensity

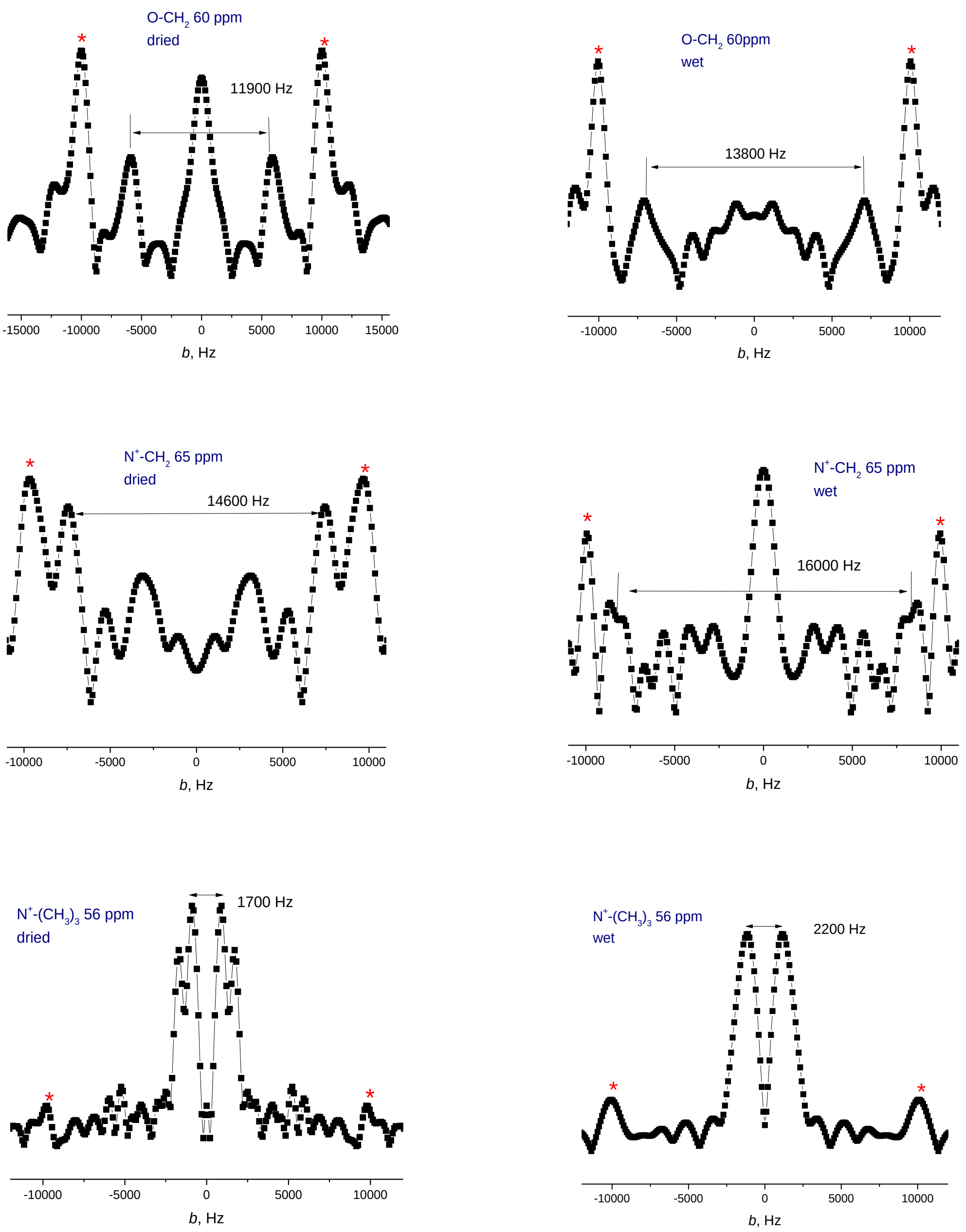

Figure S5. The Fourier transform (FT) over the time dependence of the oscillating part of CP intensity $I(t)$ apodized using a Gauss function with interactively adjusted decay[17] in wet and dried PMETAC (Figure 3) for some spin sites and the obtained dipolar splitting values. The coupling constant D_{CH} can be determined from b values, rescaling them by the factor of $\sqrt{2}$ because of the HH matching for $n = \pm 1$ was fulfilled in the present experiments. The peaks at the MAS frequency (10 kHz) are related to periodic quasi-equilibriums[S1] are marked by the asterisks (*).

The calculation of the correlation time of the I-spin bath in the rotating frame τ_X

For a dipolar interaction Hamiltonian in strong RF fields (ω_{1I} >> ω_r), the spin-diffusion rates R_{dp} and $R d_f$ can be expressed as

$$R_{dp} = \frac{N_2^{II}}{3}\left[\frac{2\tau_X}{1+(\omega_r\tau_X)^2}+\frac{\tau_X}{1+4(\omega_r\tau_X)^2}\right], \quad \text{(S1)}$$

$$R_{df} \approx \frac{N_2^{II}}{4}\frac{\tau_X}{1+(\omega_{1I}\tau_X)^2}, \quad \text{(S2)}$$

where $\mathcal{N}^{II}_2$ is the second moment of the dipolar fluctuation autocorrelation function.[4] In the case of PMETAC, neither the condition of the extreme narrowing ($\omega_{1I}\tau_x$ << 1) nor the strong collision limit ($\omega_r\tau_x$ >> 1) are fulfilled. Therefore, no asymptotic relations were used, and τ_X was calculated from eqs S1 and S2 by the rigorous solving. The results are presented in the paper in Figure 6.

Effect of angular averaging on the spin-diffusion rates

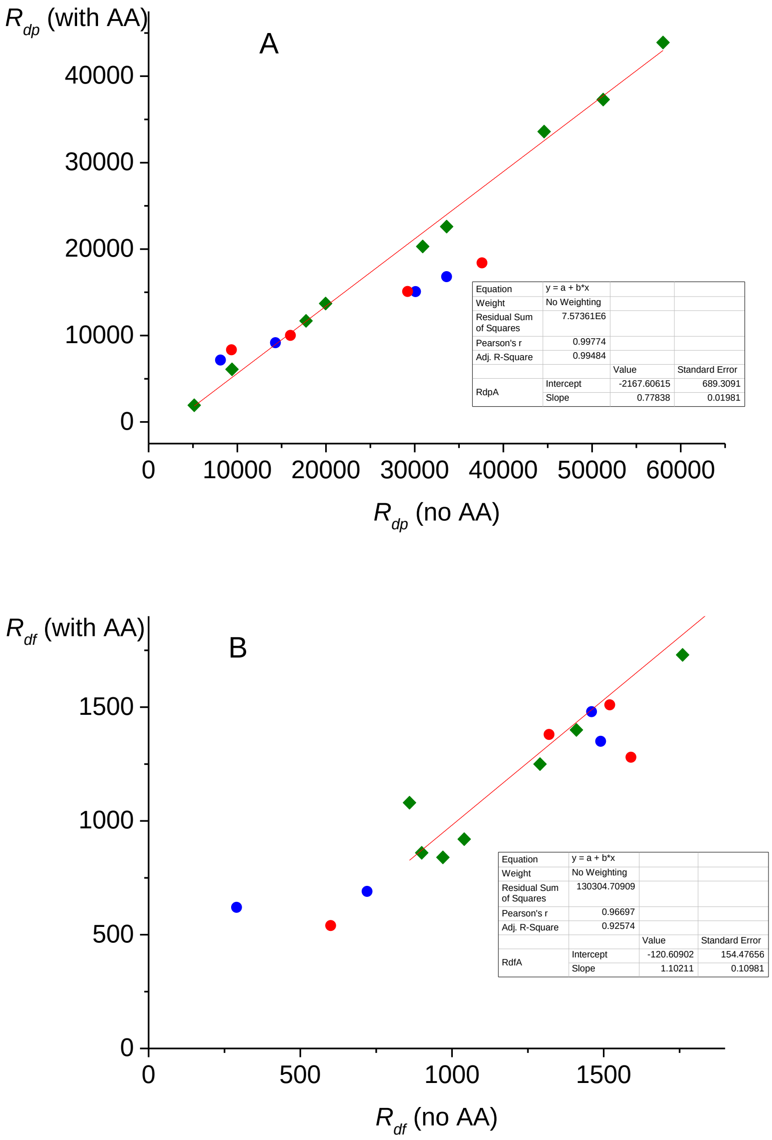

Figure S6. The correlations between the R^{I}_{dp} (A) and R^{Σ}_{df} (B) rates deduced for all experimental kinetics applying the angular averaging (AA) and without AA.

The processing of impedance spectroscopy data

The dielectric relaxation was approximated using Cole – Cole function:

$$\varepsilon^* = \varepsilon_\infty + \frac{\Delta\varepsilon}{1+i\omega\tau^{1-\alpha}}, \quad \text{(S3)}$$

where, α is the parameter determining the breadth of distribution of relaxation times, ε_∞ is the high frequency dielectric permittivity, τ denotes the relaxation time, ω is the angular frequency and $\Delta\varepsilon$ is the contribution of the process to dielectric permittivity.

The real part of conductivity σ' was calculated as

$$\sigma' = \omega\varepsilon_0\varepsilon''(\omega), \quad \text{(S4)}$$

where ε_0 is the dielectric permittivity of vacuum. According to the Jonscher power law, σ' is related to the direct current conductivity σ_{DC}:

$$\sigma' = \sigma_{DC} + A\omega^S, \quad \text{(S5)}$$

where A and S are the constants. The σ_{DC} values were estimated using eq 5S and the experimental set of the frequency dependences of conductivity at various temperatures. The dependence of σ_{DC} on temperature is presented as Arrhenius plot in Figure 5S.

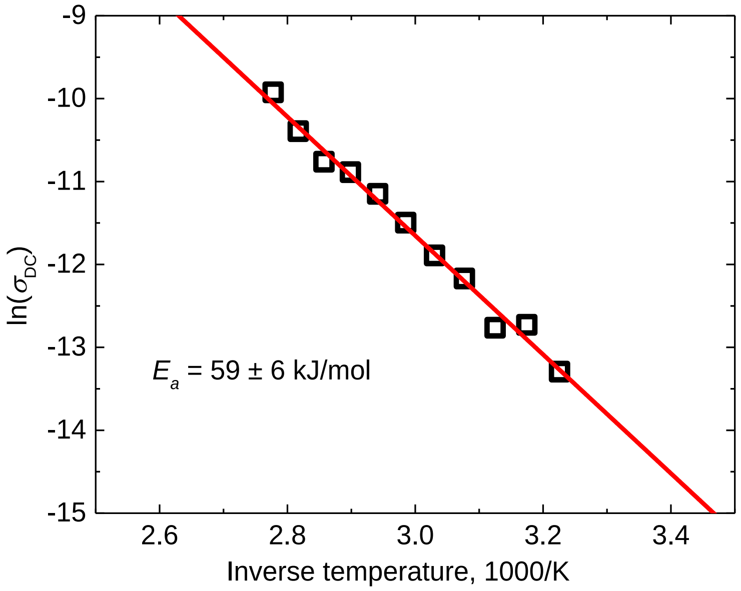

Figure S7. The temperature dependence of direct current conductivity of PMETAC in the Arrhenius plot.

REFERENCES

(S1) Sakellariou, D.; Hodgkinson, P.; Hediger, S.; Emsley, L. Experimental observation of periodic quasi equilibria in solid-state NMR. *Chem. Phys. Lett*. **1999**, *308*, 381–389.

The complete files of the experimental and processing data are available from the corresponding author on reasonable request.
